# Recent Developments in AI and ML for IoT: A Systematic Literature Review on LoRaWAN Energy Efficiency and Performance Optimization

**DOI:** 10.3390/s24144482

**Published:** 2024-07-11

**Authors:** Maram Alkhayyal, Almetwally Mostafa

**Affiliations:** 1 Department of Information Systems, College of Computers and Information Sciences, Princess Nourah Bint Abdulrahman University, Riyadh 11564, Saudi Arabia; 2 Department of Information Systems, College of Computers and Information Sciences, King Saud University, Riyadh 11543, Saudi Arabia

**Keywords:** IoT, LoRaWAN, ML, AI, sensors, resource management, energy efficiency, network performance

## Abstract

The field of the Internet of Things (IoT) is dominating various areas of technology. As the number of devices has increased, there is a need for efficient communication with low resource consumption and energy efficiency. Low Power Wide Area Networks (LPWANs) have emerged as a transformative technology for the IoT as they provide long-range communication capabilities with low power consumption. Among the various LPWAN technologies, Long Range Wide Area Networks (LoRaWAN) are widely adopted due to their open standard architecture, which supports secure, bi-directional communication and is particularly effective in outdoor and complex urban environments. This technology is helpful in enabling a variety of IoT applications that require wide coverage and long battery life, such as smart cities, industrial IoT, and environmental monitoring. The integration of Machine Leaning (ML) and Artificial Intelligence (AI) into LoRaWAN operations has further enhanced its capability and particularly optimized resource allocation and energy efficiency. This systematic literature review provides a comprehensive examination of the integration of ML and AI technologies in the optimization of LPWANs, with a specific focus on LoRaWAN. This review follows the PRISMA model and systematically synthesizes current research to highlight how ML and AI enhance operational efficiency, particularly in terms of energy consumption, resource management, and network stability. The SLR aims to review the key methods and techniques that are used in state-of-the-art LoRaWAN to enhance the overall network performance. We identified 25 relevant primary studies. The study provides an analysis of key findings based on research questions on how various LoRaWAN parameters are optimized through advanced ML, DL, and RL techniques to achieve optimized performance.

## 1. Introduction

IoT technologies are popular nowadays in various fields, such as smart agriculture, health, asset monitoring, security, and smart homes. There are certain conditions that are required for these technologies to operate, such as a long communication range, low energy usage, and affordability [1]. As usage of IoT devices has become more evident, tradeoffs have arisen, such as power consumption. Although extended transmission ranges are offered by mobile cellular technologies such as 2G, 3G, and 4G, there is a trade-off in these devices, namely that their batteries deplete faster [2]. There are popular short-range technologies such as BLE and ZigBee, but they are not able to fulfill the demands of long-distance communication. These devices had a high deployment cost as well as more congestion in cases of diverse deployment in a dispersed environment. This ultimate need for extended communication with enhanced resources has given rise to LPWAN technologies [3]. These technologies have emerged as a viable option for meeting the unique requirements of IoT applications. These technologies offer advantages such as extended transmission ranges, low energy consumption, and cost-effective implementation options.

LPWANs offer an affordable way for machine-to-machine (M2M) connectivity in addition to enabling and providing low-power and long-range communication [4]. These technologies can use these capabilities, and because of that, devices with battery lives of years or even decades can be deployed. LPWAN technology has paved the way for new opportunities in IoT applications, particularly in places like rural areas where cellular network coverage is limited [5]. Although LPWANs are being used in a variety of industrial sectors, it is interesting to note that the majority of public LoRaWAN gateways are in metropolitan regions [6,7]. Numerous applications are supported by these gateways, such as waste management, smart metering, weather stations, smart parking, flood monitoring, and infrastructure monitoring. The strategic placement of LoRaWAN gateways in urban environments is in accordance with the strengths and complements of the technology as well as the specific needs of these applications [8].

**Figure 1:**
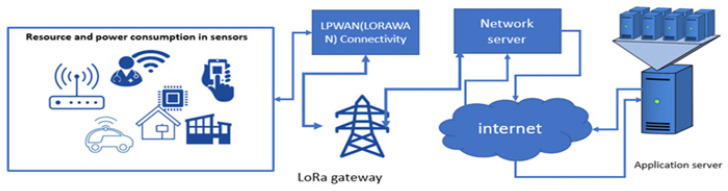
Network Architecture for LoRaWAN.

The two most widely used LPWAN applications are Sigfox and LoRaWAN, with LoRaWAN operating as an open standard for the network and a link layer above the LoRa physical layer [8]. Sigfox was the first LPWAN specifically created for the IoT back in 2019. Recent years have also seen the emergence of novel LPWAN technologies that use unlicensed radio bands for low-power, long-range transmission, such as Symphony Link, Weightless, and Ingenu RPMA. In comparison with technologies such as ZigBee and BLE, which have used mesh topologies for extended communication, LPWAN technologies like Sigfox, LoRaWAN, and NB-IoT have used a star topology in which the end devices are directly connected to public base stations [3]. This approach eliminates the need for connected end-devices to listen to the radio channel before transmitting, and this optimizes energy consumption. As base stations remain always-on, they guarantee immediate access to connected end-devices [8]. Figure 1 shows the basic network architecture for LoRaWAN.

A technical comparison of Sigfox, LoRaWAN, and NB-IoT can be done as follows.

### 1.1. Sigfox

Sigfox deploys proprietary base stations under unlicensed sub-GHz ISM bands with the help of its patented UNB (Ultra Narrow Band) technology. Sigfox can reach a maximum data rate of 100 bps by using BPSK modulation in an ultra-narrow band of 100 Hz. This novel method maximizes the use of the frequency spectrum, which leads to lower power consumption, significant receiver sensitivity, and cost-efficient antenna design [9].

In the initial days of its release, Sigfox was limited to only uplink transmission. Later, it was able to provide bidirectional communication, where downlink transmission occurs after an uplink broadcast. With a payload length of 12 bytes, the maximum number of uplink messages that may be sent in a day is 140. All uplink communications cannot be acknowledged by downlink messages. The payload was restricted to four per day. Sigfox uses transmission duplication and time/frequency diversity to ensure the reliability of uplink transmission without acknowledgment. Three separate frequency channels are used by each end-device to transmit messages, which reduces costs and simplifies the complexity of the end-device [8].

### 1.2. LoRaWAN

LoRaWAN has been standardized by the LoRa Alliance since 2015, and it uses LoRa spread spectrum technology in the unlicensed sub-GHz band. To adapt the tradeoffs between range and data rates, LoRaWAN has six spreading factors (SF7 to SF12), and it offers data rates between 300 bps and 50 kbps. The maximum payload length for each message is 243 bytes. LoRaWAN adopts a unique approach where each message transmitted by an end-device is received by all of the base stations in range [5]. This method enhances the ratio of communication reliability. LoRaWAN provides multiple communication classes that address different latency requirements. These classes are [8]:

- Class A (bidirectional end-devices): This class has the lowest power consumption for applications requiring short downlink communication after an uplink message.
- Class B: This class introduces scheduled receive slots in addition to random windows and enhances latency predictability.
- Class C: This class continuously opens extra receive windows, suitable for IoT applications with continuous energy and power resources.

LoRaWAN’s ability to support multiple communication classes and exploit redundant reception for localization provides flexibility in meeting diverse IoT application needs.

### 1.3. NB-IoT

A LPWAN technology called narrowband-IoT (NB-IoT) coexists in licensed frequency bands with GSM or LTE [10]. NB-IoT provides a range of operating modes, such as standalone, guard band, and in-band operation, within a 200 KHz frequency bandwidth [11]. It uses orthogonal frequency-division multiple access (OFDMA) in the downlink, FDMA (Frequency Division Multiple Access) in the uplink, and QPSK modulation [12]. NB-IoT reduces LTE functionalities to the minimum amount that is required for IoT applications based on the principles of the LTE protocol. With a maximum downlink throughput rate of 200 kbps and an uplink throughput rate of 20 kbps, it permits over 100,000 devices to be connected per cell. It has 1600 bytes of maximum payload size [8].

### 1.4. IoT Factors to Consider

Several variables must be considered when choosing an LPWAN technology for an IoT application.

Scalability and length of payload are essential factors. While all three technologies are very scalable, NB-IoT performs more effectively, with more than 100,000 devices per base station. NB-IoT also supports payload lengths up to 1600 bytes, which is longer than Sigfox (12 bytes) and LoRaWAN (243 bytes). The decision is based on the requirements of the application.

Considering factors such as coverage and range, Sigfox performs better with a range that exceeds more than 40 km, and it is suitable for entire city coverage with minimal base stations [8]. NB-IoT has the lowest range, typically less than 10 km, targeting locations not covered by cellular mobile networks. In comparison, LoRaWAN offers a similar, yet slightly extended, range of up to 20 km.

While Sigfox and LoRaWAN ecosystems are developed and commercially accessible, NB-IoT is still in the process of expanding internationally [5]. NB-IoT could use more energy with synchronous communication, which might reduce the lifetime of the device. LoRaWAN-Class-C and NB-IoT are recommended for low-latency applications [4]. Sigfox and LoRaWAN prioritize energy conservation and enhance battery life with end-device sleep modes. On the other hand, synchronous communication in NB-IoT may result in increased energy consumption and a lower device lifetime. While Sigfox and LoRaWAN-Class-A serve latency-insensitive applications, LoRaWAN-Class-C requires energy efficiency (EE) tradeoffs to achieve low bidirectional latency [8].

An overview of the technical aspects and performance characteristics of Sigfox, LoRaWAN, and NB-IoT is given in Table 1.

Intensive research has been done in the past about LoRa and LoRaWAN technology and its resources. This survey offers an in-depth study of various LoRaWAN resource features such as spreading factor, Bandwidth, Transmission power, and energy consumption. The study offers insights into the recent developments of LoRaWAN technology, considering factors such as network performance and stability. The study explores advanced strategies for optimizing EE by discussing state-of-the-art methods. The study investigates the integration of ML and AI algorithms to optimize LoRaWAN performance. Scaling LoRaWAN networks for extensive IoT deployments is also explored, considering key trends and challenges. The survey also identifies gaps and opportunities for the future, specifically focusing on performance enhancement and EE optimization.

The rest of the survey is organized as follows. Section 2 offers the methodology used to gather the state-of-the-art information along with the research questions formulated. The properties and most relevant algorithms of LoRa and LoRaWAN technologies are discussed in Section 3. Section 4 presents the state-of-the-art with LoRaWAN and its integration with ML. Section 5 discusses the implications and analyzes the literature review based on research questions. Section 6 presents the conclusion and future work.

## 2. Methodology

This systematic literature review (SLR) has followed the PRISMA 2020 guidelines [14] to comprehensively explore the current state-of-the-art in LoRaWAN technology. The SLR is focused on network performance, stability, EE, and future trends. The refined search string has been applied to reputable platforms, and initially, 158 results were identified. After applying extensive filters and eliminating duplicates, we obtained 161 relevant papers. Subsequent analysis and application of exclusion criteria resulted in the inclusion of a final set of 25 papers.

### 2.1. Rationale and Objectives of SLR

The SLR addresses the necessity of understanding the latest developments in the field of LoRaWAN technology as well as its integration with ML to optimize network performance, stability, and EE. The objective of this review is to conduct a comprehensive SLR to gain valuable insights into LoRaWAN technology, its challenges, and future trends.

### 2.2. Scope of Study and Research Questions

The scope of the study is to provide an in-depth analysis of the LoRaWAN technology and its integration with ML. The study also provides a comprehensive understanding of the state-of-the-art ML algorithms, network simulators, and datasets that are acquired by the authors to optimize resource consumption in LoRaWAN technology. To identify the scope of the study, we have formulated the following research questions:

1. What state-of-the-art methods exist for optimizing LoRaWAN energy efficiency and enhancing network performance?
2. How can ML and AI algorithms be used to optimize resource constraints to enhance energy efficiency and LoRaWAN’s performance?
3. What are the parameters that are optimized to enhance the performance of LoRaWAN networks?
4. What gaps and opportunities exist for future research in the fields of LoRaWAN performance and energy efficiency optimization?

### 2.3. Eligibility Criteria

The explicit exclusion and inclusion criteria are established to ensure that only relevant research material is included in the SLR.

#### 2.3.1. Inclusion Criteria

Inclusion criteria are established to ensure that only relevant studies are included in the SLR. Selected studies were required to be directly related to LoRaWAN technology and specifically involve the integration of ML within the context of LPWAN technologies. This criterion has ensured that the studies that are selected are aligned with the primary objectives of the review, which explore the integration between LoRaWAN and ML.

#### 2.3.2. Exclusion Criteria

The exclusion criteria were designed to filter out studies that did not align with the core focus of the SLR. The studies that were excluded are those with irrelevant abstracts or book chapters that are out of the study’s scope. The studies that only concentrated on LPWAN without integrating ML were also excluded, as were those that only addressed general LPWAN and LoRaWAN resource optimization without specific ML integration. These criteria have helped in maintaining the precision and relevance of the included studies.

### 2.4. Information Sources

A systematic search was conducted across multiple databases, and the articles were collected from reputable sources and platforms such as Science Direct, IEEE Xplore, MDPI, and Google Scholar. The search string (“LoRaWAN Network Performance” OR “LoRaWAN Stability”) AND (“Energy Efficiency” OR “Enhanced Lifetime”) AND (“Machine Learning” OR “Artificial Intelligence” OR “AI Algorithms” OR “Performance Optimization”) AND (“Scaling LoRaWAN Networks” OR “Emerging Trends” OR “Network Challenges”) AND (“Future Research” OR “Research Gaps” OR “Opportunities”) was applied, and results were filtered.

### 2.5. Results

**Figure 2:**
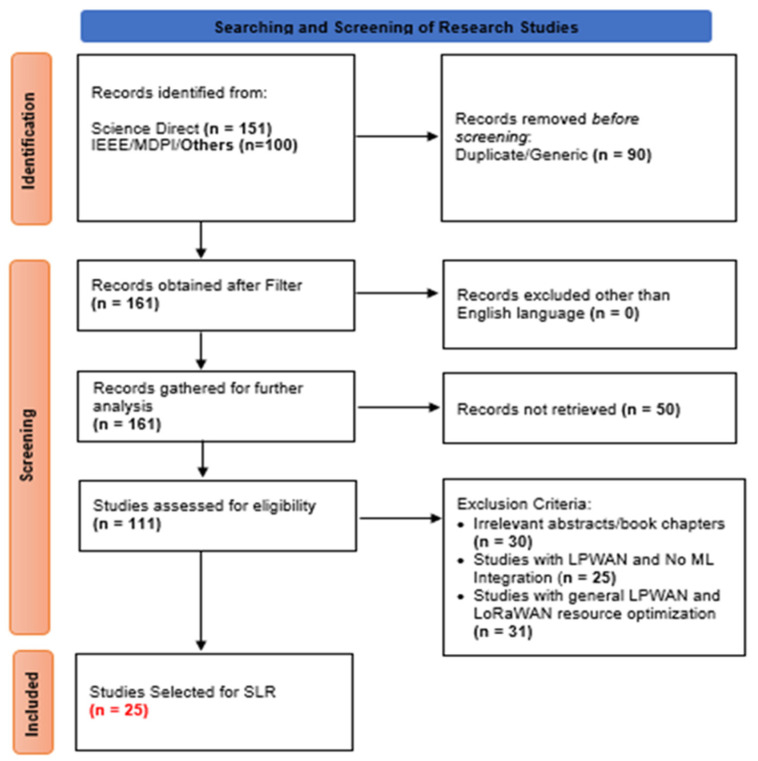
SLR Methodology, PRISMA 2020.

The complete selection process and results of the search are presented in the form of a flow diagram, as shown in Figure 2, which indicates the number of records identified, screened, and included in the final phase. A complete analysis of the research questions has been provided, and a general interpretation of the results is also given in the review process.

### 2.6. Contribution of the SLR

**Figure 3:**
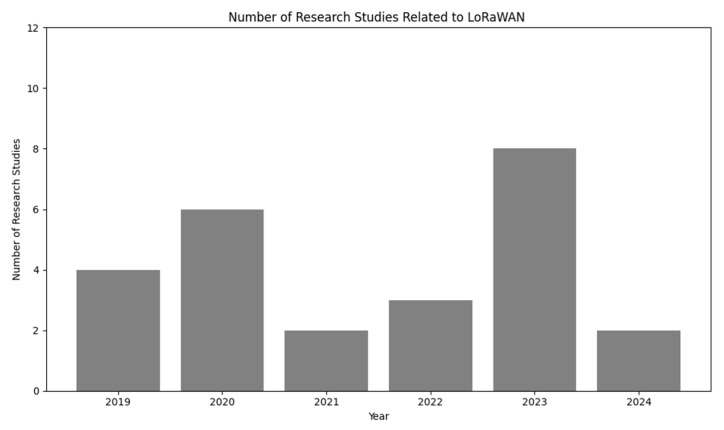
Selection of Studies with respect to each year.

The contributions of the study can be stated as follows:

1. The study provides comprehensive insights into recent advancements in LoRaWAN technology in terms of ML algorithms, simulations, and datasets.
2. The study provides an extensive evaluation of innovative methods for energy-efficient LoRaWAN operation.
3. The study explored the integration between ML, AI, and LoRaWAN.
4. The study gives an in-depth analysis of scalability challenges and trends in LoRaWAN networks.
5. The study has identified the research gaps and future opportunities for advancements in LoRaWAN.
6. The number of studies selected with respect to each year is shown in Figure 3.

## 3. Properties and Characteristics of LoRaWAN Technologies

There are several aspects of the LoRaWAN characteristics that have an impact on the overall performance of the network. These properties are discussed in this section.

### 3.1. Modulation Technique

The modulation methods that are supported by LoRaWAN are FSK and LoRa [15]. Chirp spread spectrum (CSS) modulation serves as the foundation for LoRa modulation, which can operate in a variety of spreading factors (SF) that allow a trade-off between transmission range and data throughput. LoRa uses an advanced modulation technique that enables low-power, long-range transmissions. LoRa modulation is resistant to fading and interference by design [16]. The signal is modulated onto a chirp waveform in LoRa modulation, which is a tone that changes in frequency in a linear manner over time. LoRa signals may be decoded even at a negative signal-to-noise ratio (SNR) because of the energy capture effect, which occurs as the receiver gradually gains more energy over time [17]. The architectural components of the modulation are mapped to the LoRaWAN protocol stack. A preamble or time-frequency synchronization sequence is transmitted by a device to determine the free channel capacity in LoRaWAN. The device then uses the measured channel capacity to select the correct SF. The selected SF is used to send the data payload in the next step. Another modulation method that LoRaWAN supports is FSK modulation with a maximum data rate of 50 kb/s. Short-range communications use FSK modulation, which benefits mostly from its low complexity. Binary data is communicated via FSK modulation, which shifts the carrier signal’s frequency between two levels. The frequency difference between the modulating and carrier signals determines the data rate of the FSK signal [18].

In certain regions, LoRaWAN also supports Long Range Frequency Hopping Spread Spectrum (LR-FHSS), which offers a reliable and robust connection technique. A pseudo-random hopping sequence over a group of sub-carriers is the foundation of LR-FHSS, which reduces the impact of interference [17].

### 3.2. Data Rate and Payload

The maximum payload length and related data rate in LoRaWAN are significantly affected by the interaction of spreading factors, bandwidth, and coding rate. In general, choosing lower bandwidths and spreading factors increases transmission ranges but decreases data rates; on the other hand, choosing lower bandwidths and spreading factors increases data rates but reduces transmission ranges [19].

Various factors influence the maximum payload length and data throughput. These factors include error correction, frame format, and other variables that may change throughout LoRaWAN networks and devices. The LoRaWAN specification describes data rates for 125 kHz bandwidth that range from DR0, which is the least resilient and considered the highest data rate, to DR5, which is the most robust and considered the lowest data rate, and for 500 kHz bandwidth, which vary from DR6 to DR7. Payload lengths range from 222 bytes (DR0 to DR2) to 51 bytes (DR3 to DR5) and 1152 bytes (DR6 and DR7) [20]. LoRa data rates are not limited to any specific range, but they can range from 0.162 kb/s to 50 kb/s. Depending on the deployment location, LoRaWAN-based systems using LoRa in the physical layer (PHY) can achieve data speeds of 5.47 kb/s as well as 11 kb/s, or 21.9 kb/s [18]. The frequency shift keying (FSK) modulation technique enables the LoRaWAN to achieve a maximum data rate of 50 kb/s [21].

To achieve an extended range and less inference, it is important to balance considerations such as lower data rates with higher spreading factors or higher data rates with lower spreading factors to improve throughput and overall data transmission. The choice can be according to the requirements of each application or device, which offers an optimal trade-off between data transmission efficiency and network performance [21].

### 3.3. Coding Rate (CR)

CR is considered an important LoRaWAN characteristic that has an impact on both transmission rate and error correction performance. There are six CR values that are supported by LoRaWAN; these include 4/5, 4/6, 4/7, 4/8, 2/3, and 4/5. These values determine the ratio of redundant bits needed for error correction. Higher CR values enhance error correction but extend the time on air, while lower values optimize transmission efficiency but compromise error correction. For instance, CR value 4/5 means that 1 in 5 bits is for error checking [3]. The higher coding rates, such as 4/5 and 4/6, offer high rates in terms of throughput, but they also offer less resistance to nose and inference. Meanwhile, the lower coding rates, such as 4/7 and 4/8, offer more resistance, but the throughput rate is lower, which shows the potential tradeoff [22].

The choice of CR depends on application needs as well as interference levels and available bandwidth, with careful consideration, which can further involve testing and simulation for optimal deployment. LoRaWAN devices must transmit data at constant coding rates in both the uplink and downlink directions [21].

### 3.4. Spreading Factor

In LoRaWAN, spreading factors (SF) are critical in establishing an optimal trade-off between data throughput and transmission range. LoRaWAN offers six spreading factors, which range from SF7 to SF12, that allow adaptability to specific application requirements. Higher SF values, such as SF12 [23], provide coverage ranges of up to 45 km in rural regions and more than 5 km in urban settings, but at a cost to the data rate. The number of chips required for representation and the length of each symbol are determined by the spreading factor. A lower SF results in shorter symbol duration and higher data rates, but reduced resistance to noise. Higher SF values, on the other hand, improve interference resistance but result in lower data rates [20].

The choice to balance the intended data rate and range according to the requirements of the application is based on preference and environment. All base stations within a certain range receive every message from an end device (ED), which enhances the communication reliability of LoRaWAN. However, this requires the setup of several base stations, which raises the cost of network infrastructure. Spreading factor values are usually set between 7 and 12, and their modification provides a flexible way to handle transmission distance as well as data rate and energy consumption [24]. This allows it to be used in a variety of situations, such as high-attenuation long-range communications and environments with high interference. Thorough testing and optimization based on specific use cases produce the ideal spreading factor, which is essential for effective performance [25].

The nominal bitrate (R_bit_) in bits per second can be expressed mathematically as seen in Equation (1) using the parameters (Spreading Factor—SF, Bandwidth—BW, Coding Rate—CR) as follows [26]:

(1) $$R_{bit}=SF\cdot \frac{BW}{2^{SF}}\cdot CR$$

This formula considers the Spreading Factor (SF) and Bandwidth (BW) to calculate the bitrate, incorporating the Coding Rate (CR) in the numerator. The Spreading Factor contributes to the duration of each symbol, while the Bandwidth influences the frequency range. The Coding Rate determines the encoding efficiency that impacts the time on air for data transmission. The formula in Equation (1) shows the relationship between these parameters in determining the nominal bitrate in LoRa communication [20].

### 3.5. Carrier Frequency (CF) or Frequency Band

LoRaWAN operates in free frequency bands such as 433 MHz, 865 MHz, and 915 MHz, which eliminates the requirement for a license to operate. These frequency bands serve as the communication medium for LoRa nodes and LoRa gateways within the LoRaWAN network [27]. The network layer protocol functions on the medium access controller (MAC) and enhances an impressive range of approximately 10 miles or 15 km. LoRaWAN has a default bandwidth of 125 kHz for wireless transmission on specified frequencies. The choice of bandwidth, which can be according to the frequency plan and lie anywhere between 125 kHz and 500 kHz, also impacts the amount of time it takes to modulate a data packet [19]. The LoRa modules have stated transmission powers of 13 dBm, 16 dBm, and 20 dBm at 433 MHz, 868 MHz, and 915 MHz, respectively [28]. LoRa follows standard protocols that manage the duty cycle, which regulates how long a device is active during transmission. There are specific values that can be specified for this option, including 0.1%, 1%, or 10%. Furthermore, several countries have restrictions regulating the Time on Air (ToA) for packets carried using LoRa. Regulations use mechanisms like duty cycles, dwell times, and Listen Before Talk (LBT) to make sure people follow the rules [18]. In several regions, the commonly used frequency bands are subdivided into specific frequency ranges, each allocated for distinct purposes, such as indoor or outdoor use, with corresponding power limits. The frequency bands are also deployed in certain regions, depending on regulatory constraints, the available spectrum, and interference considerations. The choice of a specific frequency band is based on a critical decision, which can be influenced by these factors to ensure optimal performance and compliance [21].

### 3.6. Adaptive Data Rate (ADR)

Adaptive Data Rate (ADR) is also an important LoRaWAN approach that maximizes battery life for static end devices and improves overcommunication efficiency. This method is based on real-time channel conditions, and therefore it dynamically modifies three crucial parameters, which are SF, CR, and Transmit Power (TX). ADR becomes essential when channel requirements are dynamic and are faced by variable interference, signal quality, and propagation conditions [20].

Since ADR regularly checks the integrity of the transmission link, its adaptive nature makes it useful for devices that are mobile in nature and navigate in dynamic settings. The network server can initiate downlink messages that prompt data rate adjustments to correspond to the present conditions. The network server uses metadata such as SNR, Received Signal Strength Indicator (RSSI), timestamp, and signal quality to decide which SF configuration is best for each device. ADR considers key factors such as the link budget of a device to carefully adjust SF configurations according to the signal strength that is available. There is a difference between network-managed ADR and Blind ADR. Network-managed ADR is implemented by a server for fixed positioned end devices, while Blind ADR autonomously implements the mobile end devices, as they already have knowledge of the current link state [18].

## 4. Machine Learning and LoRaWAN

LoRaWAN technology may be enhanced in several ways with the use of ML, which enhances resource efficiency and optimization. Energy Consumption Optimization is one important field where machine learning can show immense benefits. ML algorithms can evaluate and optimize device usage in LoRaWAN by exploring complex data patterns. This optimization promotes resource-efficient and sustainable operations by extending the battery life of devices as well as reducing the need to replace them more often [29].

In network management, ML techniques also play an important role. These algorithms can predict potential faults and optimize network settings through continuous performance analysis of the network. This approach reduces the potential challenges before they escalate. Proactive management improves productivity as well as making LoRaWAN robust and reliable [30]. Another field where ML has a significant influence is traffic prediction and load balancing. ML can predict traffic patterns on the LoRaWAN network by using predictive models [31]. This prediction enables effective load distribution across LoRaWAN gateways, ensures optimal utilization of network resources, and prevents bottlenecks. ML algorithms can identify problems in the LoRaWAN network through fault detection and localization. As a result, errors can be fixed, downtime is reduced, and network performance is enhanced [18].

The current literature is comprised of various ML model techniques and datasets that are used to enhance resource and EE optimization in LoRaWAN. This section covers various ML and AI-based techniques, such as supervised and unsupervised learning techniques, along with DL (Deep Learning), Neural Network (NN), and Reinforcement Learning (RL).

### 4.1. DL and NN Techniques and Models for LoRaWAN Performance Efficiency

To maximize the energy efficiency (EE) in LoRa networks, the study [32] has proposed a DL approach that uses artificial neural networks (ANNs). The authors studied the optimal transmit power for maximization of EE. In their methodology, the authors have trained the ANNs twice with two datasets. First, the authors have used model-based data, and second, they trained the model with a real-world dataset that was obtained by Monte Carlo simulations. The study used the Poisson Cluster Process for stochastic geometry modeling for optimization of parameters such as SF, CR, and BW. Simulations are implemented in MATLAB, while real-world data training is performed with Microsoft Azure. Results demonstrated that ANNs have performed better with negligible gaps between partial and full optimum architectures. It was also observed that there is a possibility of freezing some layers during retraining without facing a major decline in performance. The study [33] proposed a novel deep learning-based resource allocation strategy using a Gated Recurrent Unit (GRU) to address resource management issues in large-scale LoRa-enabled device deployments. The GRU-based method predicted and reduced packet loss caused by incorrect SF utilization. It solved the issue that occurred with LoRaWAN’s ADR mechanism, which experiences difficulty adapting to sudden shifts in propagation settings. The technique pre-trains a GRU model to assign suitable SFs by combining offline and online modes, which results in an 11% increase in packet success ratio. The reason the authors chose GRU over other ML models, such as LSTM and SVR, was because it could adjust to different input sequence lengths. The suggested approach has shown better performance when compared with current techniques, and experiments were carried out in Python using TensorFlow and Keras frameworks with data from a simulated LoRaWAN network. This study [34] has addressed the issue of packet loss caused by inefficient use of SFs. The authors stated that current resource allocation approaches such as ADR and BADR are not sufficient to address the problem of massive packet loss. The authors presented a novel approach, AI-ERA, that is based on a DNN-based resource allocation strategy. AI-ERA improves packet success rates (PSRs) for both stationary and mobile IoT applications by using AI to predict optimal SFs based on dynamic channel conditions. AI-ERA maximized the PSR through offline training and online prediction modes and used the NS-3 simulator. The proposed strategy has allocated the predicted SFs to end devices in a proactive manner. The results showed that AI-ERA is more effective at improving SF management than traditional ADR techniques.

The study [35] has proposed an ML technique based on NN to enhance the accuracy of indoor localization within the LoRaWAN network. The authors stated that there is a primary challenge in the variability in signal strength that is caused by environmental factors, and this causes more energy to be consumed by devices. Their proposed solution is based on the train-then-test methodology, where supervised learning is utilized to build a ML model for each indoor location area. The authors optimized TP, CR, SF, and BW parameters by selecting the minimum values that still meet the required accuracy level for device localization. They have predicted the optimal values of these parameters by using machine learning-based signal strength prediction that has reduced energy consumption while maintaining an accuracy of 98 percent.

### 4.2. Reinforcement Learning (RL) Techniques

This research study [36] addressed the distribution of network resources, such as SF and TP, in a LoRaWAN. The study aimed to minimize TP with respect to the highest possible data rate. The authors increased network efficiency by using DRL, which created multiple agents that would identify the most optimal transmission parameters for terminal nodes. The authors focused on throughput with respect to energy transmission and compared their proposed approach with the current LoRaWAN’s ADR MAX using the NS3 simulator. Findings suggest that the proposed approach has improved throughput by around 15%. DRL agents that were trained to predict optimal values based on network circumstances were also used to optimize SF and TP. This technique aims to reduce transmission power while balancing throughput and reliability. The network may continue to transmit data efficiently while using less energy through the dynamic adaptation of SF and TP. The study [37] aimed to develop an adaptable LoRaWAN strategy that is appropriate for industrial applications by using RL. The authors have proposed a novel method for optimizing LoRaWAN’s ADR function to increase the reliability of LoRaWAN networks. The authors have prioritized parameters such as data extraction rate (DER) to improve DER without compromising LoRaWAN standards. Results indicated that around 10% improvement has been observed in DER while evaluated in scenarios with high node density. The study has used SF as an action parameter and used a stochastic discrete approach to achieve optimization of SF and transmission power, which has collectively improved DER and enhanced reliability. The authors conducted extensive simulations using the Python-based simulation tool LoRaEnergySim (LES), which allows the evaluation of LoRaWAN performance under different scenarios. To maximize EE in LoRaWAN, the study [38] proposed a Markov Decision Process model that evaluated node lifespan by considering variables such as transmission energy, channel conditions, and packet size. The authors have adopted RL, specifically Deep Q-Network (DQN), to optimize the durability of nodes. The proposed method was applied to modify factors such as SF, DR, CR, and transmission power level. The model adapted the parameters, which were based on distance from the gateway and expected packet length, to overcome the constraints of transmission range and channel quality. This method significantly increased EE by allowing nodes to operate for extended periods of time without running out of battery power. The LoRa network simulator has used OpenAI Gym as a toolkit, which is widely used for research in RL to replicate the topology and state of the network.

The paper [39] presented an optimization strategy to improve network reliability for connected objects on LoRaWAN networks by utilizing federated reinforcement learning (FRL) and network slicing (NS). The model focused on optimizing device throughput by determining the best SF. Multiple agents operate together by using FRL to develop a global policy for network slices, which dedicates resources to each independent slice for throughput optimization. The authors have used MATLAB and Castalia simulators. A neural model with two hidden layers is used for Deep Reinforcement Learning, aiming for convergence to an optimal solution within a feasible training time. In terms of CR, the study maintained it at a constant 4/5 across all network slices, which is chosen for its balanced trade-off between data rate and transmission reliability, and its impact on performance has not been addressed in the study. The authors have used a dataset that included parameters such as collision rate, energy consumption, throughput, delay, and data extraction rate, as well as performance metrics such as packet loss rates and SNR across different network slices. The study noted that a 3% rejection rate for the Low Critical Latency and Efficiency (LCLE) slice has been obtained. Results indicated that throughput has been maximized for different slice types, such as Ultra High CLE and Ultra Low CLE. The study [40] addressed the problem, which concerned the dissemination of optimal network configuration parameters to end nodes in LoRa-based networks to improve per-node throughput. The authors used an RL technique to prescribe optimum distribution strategies to overcome regional restrictions on ISM band utilization. They used an RL approach, which presented a solution that formulated the optimal disseminating policies to update the parameters of LoRa-based networks. They also used deep reinforcement learning, Q-Learning, and SARSA, and the updating process has been formulated as an RL problem. The study has focused on optimizing SF and CR to improve per-node throughput. The proposed RL approach demonstrated effective optimization of SF and CR parameters and achieved up to a 147% increase in throughput. The validation and testing of models were conducted using NS-3, EDSim, and simulation modules that were integrated into MATLAB. The study [41] addressed the approach for enhancing EE in wireless underground sensor networks (WUSNs) combined with LoRaWAN technology. The authors suggested an approach that has optimized transmission topologies for underground sensors by using Multi-agent Reinforcement Learning (MARL) by considering factors and variables such as packet collisions, energy usage, and network quality. The algorithm adapts to dynamic underground environments by introducing a reward mechanism, which has enhanced its effectiveness. The authors have conducted experiments on several underground locations and network configurations, and the findings of the study showed that the MARL algorithm has optimized EE more effectively than conventional ADR techniques. The researchers have evaluated and trained the MARL algorithm on actual farm data from Ward Farm and TOSSIM, which is a simulator for TinyOS-based networks.

### 4.3. Supervised and Unsupervised Learning Techniques

The study [31] analyzed the use of ML algorithms to predict the inter-arrival time of IoT packets. To improve LoRaWAN scalability and network performance, key ML methods were used, which included k-means clustering, Long Short-Term Memory (LSTM), NN, and Decision Trees (DTs). The authors address issues with high error rates on some devices by using profiling methods that produce predictions even when training data is not available. The study analyzes the effect of spreading factors (SF) on LoRaWAN network performance and evaluates several prediction models to estimate the SF. The findings show that for 77% of actual sequences, the inter-arrival timings could be predicted with a 3.5% error. This methodology involves packet processing, device profiling through clustering, and feature extraction, which is focused on Inter-arrival time and an ML pipeline. The study adopted a combined approach that combined supervised and unsupervised learning. The authors used the real-world dataset that was gathered from water metering end-devices (EDs) over a LoRaWAN network. The dataset contained more than 4 million packets. The collection includes 18 time series characteristics, such as payload size and inter-arrival time (IT) between packets. At a granularity of 10 min, sixteen features are gathered to represent frame counters, transmission counters, etc. The authors have implemented the algorithms on a MATLAB platform by utilizing the NN Toolbox, Statistics and ML Toolbox, and Clustering Toolbox available in MATLAB. Python-based libraries like Scikit-learn and Keras are also utilized for training and evaluating the performance of different ML algorithms. The study [42] focused on EE and addressed the challenges caused by the expansion of IoT networks, which are operating on unlicensed ISM frequency bands. The author proposed an algorithm that used LoRaWAN technology to optimize parameters such as data rate, SF, and TP while taking geographic data into account. The authors have used ML clustering techniques such as the K-means algorithm to select and deploy gateways based on end-node clustering. The authors have optimized SF in their methodology to balance communication range and data rate. The proposed method has also modified the TP according to the position of the gateways and the expected volume of traffic in the area. The paper has recommended the range of 14–20 dBm as an ideal TP range to provide effective and energy-saving coverage. The findings of the study showed that a significant decrease in EC and improved coverage have been observed in comparison to previous methods. The NS-3 simulator was used for evaluation, and the authors used geographic data from real maps for dataset generation.

The study [43] focused on the issue of power consumption, specifically within the context of the LoRaWAN protocol used for IoT devices. To increase the battery life of IoT devices, the authors proposed a method that integrated ML algorithms with the low-power capabilities of LoRa technology. The authors used Support Vector Regression (SVR) and Deep Neural Network (DNN) techniques to reduce power consumption and extend battery life for LoRaWAN devices. The authors have evaluated the proposed solution by using the LoRaSim simulator and MATLAB tools. They utilized a dataset of LoRa packets generated by the simulator. The findings showed that the ML models have performed well. The study has also optimized the parameters of the LoRaWAN protocol, such as TP, SF, and duty cycle, to enhance efficiency. The authors also used multi-armed bandit and Q-learning approaches for resource scheduling and obtained improved results.

The study [44] evaluated the impact of weather conditions on the scalability of LoRaWAN technology and emphasized the improvement of RSSI measurement accuracy. It has evaluated various statistical models, such as the conventional ARIMA, ML, and DL methods. The data set included the time series of RSSI measured by eight LoRaWAN transmitter nodes. To optimize RSSI measurements in LoRaWAN technology, the researchers used a hybrid methodology that combines statistical models (ARIMA) with ANN and SVM algorithms. The approach involved data preprocessing to extract relevant features such as RSSI values, weather parameters, and time. Significant characteristics that impact RSSI values are found by statistical modeling with ARIMA. The authors have also modeled nonlinear relationships among RSSI, weather variables, and time by using artificial intelligence techniques like ANN and SVM to improve prediction accuracy. The study [25] addressed the impact of collisions on network performance as well as the assignment of SF in LoRaWAN networks. The authors created a simulation environment and proposed a novel, smart SF assignment approach that used Decision Tree Classifier (DTC) and Support Vector Machine (SVM) techniques to evaluate SF assignment schemes. According to the study, simulations using the proposed smart SF assignment approaches produced positive outcomes in terms of packet delivery ratio (PDR). The proposed approach used a simulator to generate many LoRaWAN transmission logs for various topologies. A classifier is then trained using the generated dataset to determine the optimal SF for each network node. The features of the dataset included the X and Y coordinates of the transmission source and the SF of the transmission. The authors were able to enhance network performance by optimizing the SF assignment of nodes. The simulation was performed using the MATLAB simulator on a dataset of messages that were sent across a LoRaWAN network.

The LoRa SF allocation was optimized in the study [45], and the author presented a novel K-means clustering-based approach that enabled an adjustable SF allocation approach by taking the range of user distributions into consideration. The study concentrated on a large-scale, unconfirmed-mode class-A LoRaWAN model outage and its probability of optimization without retransmissions. The authors have conducted various simulations based on Stochastic geometry in diverse settings and demonstrated the impact of node distribution on transmission reliability. They evaluated the nodes across SFs and distance from the gateway. They compared the proposed model to baseline models and found that the average coverage probability of the network was improved by up to five points. It also substantially reduced the performance lag between the best and worst-case nodes. The worst-case efficiency was remarkably improved by 1.53 times in some deployment conditions. The study [46] addressed the issue of high packet collision rates in LoRaWAN networks, which is caused by the ALOHA protocol. The authors introduced a novel Dynamic Transmission Priority Scheduling Technique (PST) based on an unsupervised learning clustering algorithm to reduce packet collisions and enhance EE. The proposed dynamic PST approach provided two transmission modes, which are conservative and non-conservative, to enhance the EE of the network. The dynamic PST also applied the unsupervised clustering algorithm as well as the Naive Bayes classifier algorithm to determine the probability of assigning a specific transmission mode to each cluster. Simulation results showed that the proposed methodology has performed better than traditional LoRaWAN applications and improved scalability.

### 4.4. Ensemble Learning, ML, and AI Based Techniques

The study [30] addressed performance degradation in LPWAN technologies, which was caused by congestion and interference. The authors stated that rule-based approaches for assigning and adapting device parameters were found to be insufficient in terms of large-scale IoT deployments, specifically the ADR algorithm in LoRaWAN. The authors have suggested a novel approach for allocating resources in LoRaWAN by using supervised ML for transmission power allocation and reinforcement learning (RL) for SF allocation. The EXP4 method was used for SF allocation in the study, while a variation in the Lasso algorithm was used for transmission power (TP) allocation. The authors evaluated their approach by using experimental data generated from the LoRaSim python simulator for LoRaWAN IoT networks. They also used PySimulator to monitor the traffic load and evaluate the proposed performance of the algorithms. Results showed that the proposed approach demonstrated approximately ten times faster convergence than the LoRaMAB EDR and the LoRaWAN ADR algorithms.

In a LoRaWAN network, mutual interference between wireless nodes can result in packet collisions and a drop in packet delivery rate (PDR). To address this issue, the authors of the study [47] proposed a ML-based Q-learning approach to efficiently allocate channels and enhance PDR. They used the weighted sum of successfully received packets as the Q-reward when applying Q-learning to the LoRaWAN system. The gateway allocated the parameters such as packet size, Q-factor, Q-reward, and discount factor to maximize this Q-reward. The performance of the proposed approach was tested by the authors using the ns-3 simulator, in which they created a synthetic dataset of randomly positioned nodes for evaluation. MATLAB was used to assess simulation results and generate plots. The findings showed that the proposed strategy has improved average PDR performance by around 20% as compared to random resource allocation methods. The authors of the study [48] addressed the need for precise outdoor localization techniques in LPWAN technologies such as Sigfox and LoRaWAN. They proposed an approach for outdoor positioning based on ensemble learning that combines exact timestamps with nanosecond signal strength. The proposed approach aimed to improve positioning accuracy so that an accurate localization would not need additional bandwidth. They configured a combined approach called KNN-RFR, which used k-nearest neighbors (kNN) integrated with the Random Forest Regressor (RFR). Results showed an improvement of 16% and 29% for mean and median error, respectively. The authors used MATLAB, Python, and LoRaSim, along with a real-world outdoor dataset composed of LoRaWAN features. The authors stated that the proposed method offered improved scalability and lower power consumption.

The authors of the study [49] addressed the problem of efficient management of IoT devices that have different velocities in dense LoRa networks. The authors proposed the LoRaDRL algorithm to enhance the PDR and to support the mobility of end-devices (EDs). The authors also aimed to utilize the same method to reduce energy usage and collisions. LoRaDRL has used DRL and cognitive radio systems to dynamically modify the SF of EDs based on channel usage and signal-to-interference-and-noise ratio (SINR) conditions. The algorithm also used Q-learning to determine optimal actions for SF adjustment while considering factors such as SFs that are currently available, current channel conditions, and channel availability. The authors have used a dataset from the dense LoRa network in Lahore, and experiments are executed using NS-3, MATLAB, and Python. LoRaDRL demonstrated resistance to widespread frequency jamming attempts as well as a 500% improvement in PDR.

The study [50] addressed the issue of energy consumption (EC) in LoRaWAN and evaluated its efficiency using metrics such as PDR, EC, and coverage area. The authors have proposed a novel algorithm called LP-MAB, which uses the RL and Multi-Armed Bandit (MAB) techniques to configure transmission parameters centrally on the Network Server side. Although LP-MAB was developed based on the ADR algorithm of LoRaWAN, according to the authors, it has offered a more optimal data rate selection strategy for EDs by using the MAB technique. LP-MAB has adopted dynamic network conditions as well as allocated resources effectively and has selected the most optimal data rates for LoRaWAN EDs through exploration and exploitation using RL. The NS-3 simulator was used in the study to evaluate the performance of the LP-MAB algorithm through simulation results. The dataset was created using an NS-3 simulation. The findings of the study showed that the proposed approach has consistently outperformed previous approaches in terms of EC while maintaining a high PDR. It showed improved performance when compared to algorithms such as ADR, BISE, ELS, and SADR. The study also suggested that LP-MAB has good scalability, and it could perform even better with the inclusion of more EDs. The study [51] used ML to enhance LoRa network performance for industrial Internet of Things applications and aimed to improve bit error rate (BER), spectrum efficiency, received power, and outage probability. The study proposed an approach to maximize the received power by using an ANN model for received power prediction and the particle swarm optimization (PSO) technique to identify optimal LoRa configurations. The author used datasets from RSSI and SNR measurements at the LoRa gateway to create simulations by using MATLAB and the LoRaWAN simulator. The results of the simulation demonstrated significant enhancements in BER, spectral efficiency, and outage probability that showed the effectiveness of the proposed optimization approach.

The study [51] proposed machine-learning-based Combined Path Loss and Shadowing (CPLS) models to optimize energy consumption in an accurate indoor localization system for the IoT that used LoRaWAN technology. The authors have used two different types of ML models to develop an improved ADR system. They used multiple-linear-regression-based CPLS (MLR) and artificial-neural-network-based CPLS (ANNs). The authors have optimized parameters such as SF, BW, and CR through the proposed ML models. The ML models were trained on an RSSI database that consisted of values that are measured in a commercial office at 19 different locations. The results showed that models have achieved a maximum energy improvement of 43 percent. The study [52] has addressed the critical issue of EE in LoRaWAN networks through clock skew estimation and fine-grained parameter optimization. The authors introduced a novel ML-based multi-agent methodology for clock skew estimation that uses ML models to accurately estimate received RSSI and SNR based on open data sources. The authors have optimized key resources such as SF, CR, and BW. The results showed significant improvements in network size enhancement of up to 21.6%. The proposed clock skew estimation methodology also achieved a network size improvement of up to 66%.

The summary of a few authors’ work has been discussed in Table 2.

## 5. Results and Discussion

The results section presents the outcomes of the research conducted on optimizing LoRaWAN networks through various techniques and models. In this section, we analyze the findings obtained from the implementation of deep learning, reinforcement learning, supervised learning, unsupervised learning, and ensemble learning approaches. The section answers the RQs identified in Section 2 by considering the literature review, followed by a detailed examination of the optimized parameters and their impact on LoRaWAN network efficiency. Through comprehensive analysis and interpretation of the findings of the literature, this section aims to provide insights into the effectiveness of different optimization strategies in enhancing the performance of LoRaWAN networks.

### 5.1. RQ1: What State-of-the-Art Methods Exist for Optimizing LoRaWAN Energy Efficiency?

To answer RQ1, we have reviewed various studies that have presented state-of-the-art methods and techniques for LoRaWAN optimization. These included various DL, ML, and RL techniques and algorithms. Several datasets and simulators have been used by various authors in their research studies. DL and NN techniques have shown significant improvement in optimizing various aspects of LoRaWAN networks. For instance, author [32] has applied ANNs to optimize transmit power, and in [33], author utilized a GRU for dynamic resource allocation in LoRaWAN. The author [34] proposed the AI-ERA approach using DNNs to enhance resource assignment in IoT applications. A study [35] used ML with NNs to improve indoor localization accuracy within LoRaWAN networks. In terms of RL techniques, authors [36] used DRL to optimize network resources and [37] developed an adaptable RL-based strategy to optimize LoRaWAN’s ADR function. Authors [25,31] utilized supervised learning to predict optimal network parameters, and [46] introduced a Dynamic Transmission Priority Scheduling Technique based on an unsupervised learning clustering algorithm.

Table 3 provides a summary of the DL and NN techniques adopted by the authors.

#### 5.1.1. State-of-the-Art RL Techniques

RL techniques are utilized across the literature that focus on optimization of resource allocation, and EE is LoRaWAN networks. Table 4 provides a summary of the techniques used to answer RQ1.

#### 5.1.2. State-of-the-Art Supervised and Unsupervised Learning Techniques

The reviewed studies have used various supervised learning techniques to address the challenges of LoRaWAN networks. Methods such as k-means clustering, LSTM neural networks, DTs [25,31] SVR [31], and DNN [43] are used to predict IoT packet timing as well as to optimize network parameters and enhance battery life. Techniques also include advanced models such as ARIMA [44], ANN, and SVM for improving measurement accuracy and managing network configurations effectively. Table 5 presents a summary of the supervised learning techniques adopted by the authors.

#### 5.1.3. State-of-the-Art Ensemble Techniques

Various studies have explored the use of ensemble, ML, and AI-based approaches to address performance degradation, EE, and reliability issues in LoRaWAN networks. Techniques such as Ensemble Learning, DRL, and MAB algorithms have been used to improve overall network performance under conditions of congestion and interference. These approaches have demonstrated significant improvements and are summarized in Table 6.

**Figure 4:**
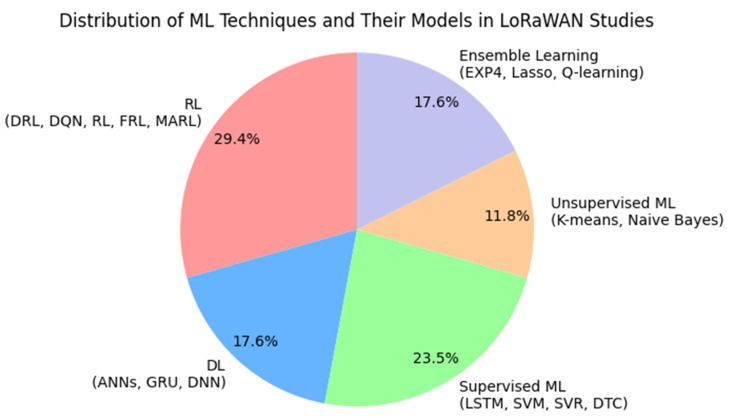
Widely adopted techniques in literature.

It has been observed from the literature that RL techniques have been widely adopted by various authors for EE LoRaWANs. DRL, DQN, generic RL, FRL, and MARL are among these novel techniques followed by DL algorithms and models. RL techniques mainly focused on optimization of network parameters, while DL techniques were used for tasks like predictive resource allocation and optimizing transmit power. Supervised learning models are applied to predict outcomes based on labeled data, such as packet arrival time. Supervised learning models were typically used for analyzing data without predefined labels, such as grouping devices based on similar behavior or optimizing transmission settings without prior knowledge of the optimal configurations. Ensemble methods combined multiple ML techniques to improve prediction accuracy, robustness, and reliability. Figure 4 presents the distribution of techniques and models in the literature.

#### 5.1.4. State-of-the-Art Datasets and Simulators

The literature section has reviewed several studies that focused on various aspects of LoRaWAN networks and highlighted how different datasets and simulators are utilized to address issues related to resource allocation, network efficiency, and optimization strategies within LoRaWAN deployments.

Table 7 shows a summary of the datasets and simulators used in the literature.

**Figure 5:**
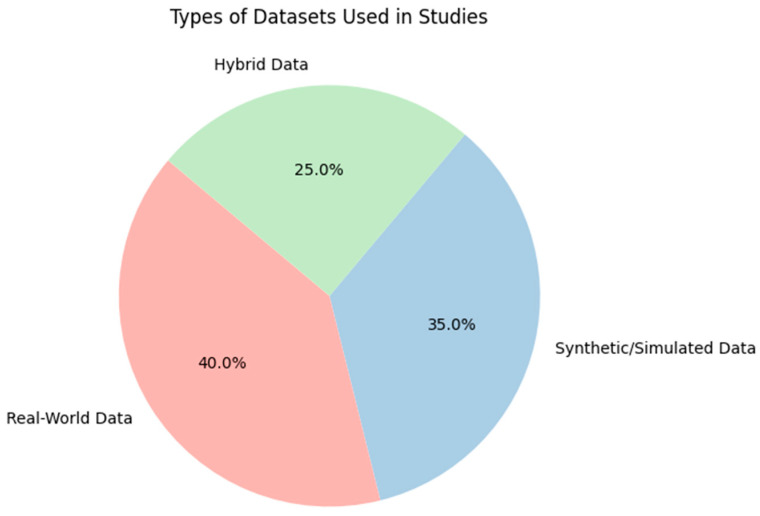
Types of datasets used in the study.

The studies often used datasets derived from both simulated environments such as NS-3 or OMNeT++ and real-world data collections. For instance, the study by [32] stated the utilization of both model-based data and real-world datasets obtained through Monte Carlo simulations to train ANNs for optimizing EE in LoRa networks. The use of MATLAB for implementing simulations and Microsoft Azure for real-world data training shows the integration of conventional programming environments with cloud-based platforms for robust data processing and simulation. In several studies [32,36,37,39,51,52] the general types of data collection parameters were mentioned, such as SNR, RSSI, and packet success rates. It was observed that in most studies [35,38,45] the specific attributes of the data, such as exact payload sizes, frequency of data collection per node, or the exact environmental variables measured, were often not mentioned. Also, the study [35] used real-world data for training machine learning models to enhance indoor localization accuracy within LoRaWAN networks. Figure 5 presents the ratio of types of datasets often used in studies.

**Figure 6:**
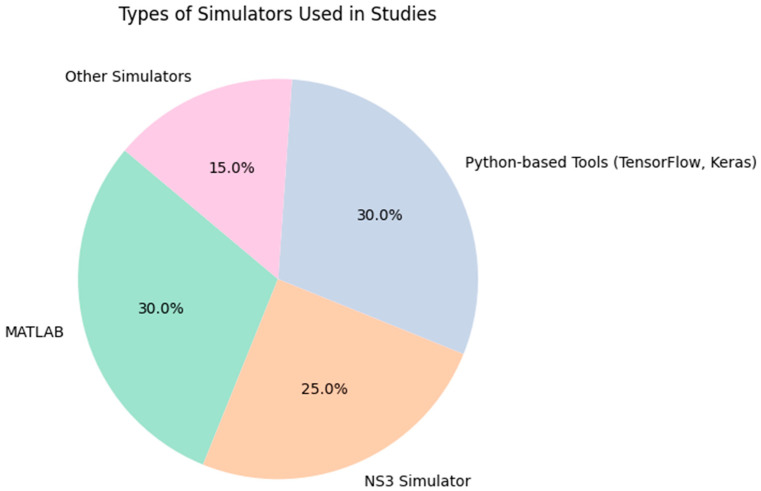
Types of simulations used in the study.

The studies [33,34] predominantly utilized the NS-3 simulator to create detailed synthetic datasets. It offers a realistic environment for testing network protocols and strategies under controlled but diverse conditions, which makes it more effective to simulate resource management strategies as explained in the studies in the literature. Some authors [35,38] have conducted simulations based on theoretical models as well as custom Python-based simulators developed specifically for their studies. Authors such as those in the study [37] have used LoRaEnergySim (LES) to simulate collided and weak packets with standard LoRaWAN data for Industrial IoT (IIoT) applications but have not explicitly discussed the size of their dataset. The integration of Python-based tools like TensorFlow and Keras for implementing DL algorithms highlighted the shift towards more sophisticated, AI-driven approaches in network management and device allocation in LoRaWAN, as shown in Figure 6. These tools enable researchers to construct and train complex models efficiently by using large datasets for enhanced predictive accuracy.

### 5.2. RQ2: How can ML and AI Algorithms Be Used to Optimize the Resource Constraints to Enhance Energy Efficiency and LoRaWAN’s Performance?

Machine learning and AI significantly enhanced LoRaWAN efficiency through adaptive algorithms such as K-Means clustering, Dynamic Priority Scheduling, and NB classifiers for predictive transmission modes, as explained in [46]. Advanced strategies such as the LP-MAB [50] further optimized the performance with a RL-based approach to minimize energy consumption and improve packet delivery. Studies [31,33,38,39,41,43,52] used DL techniques to reduce collisions and power consumption to boost network efficiency and device performance. Techniques like multi-agent systems dynamically allocate resources based on real-time conditions, as discussed in [31,43,47,48] improving scalability and network efficiency. Algorithms such as ANNs and PSO predict and fine-tune transmission parameters that have significantly enhanced network performance metrics such as SNR and BER. Methods like SVR and DNN [52] adjust transmission power and duty cycles based on data-driven predictions that extend device battery life. Ensemble methods and predictive modeling have also been discussed [48] to dynamically adjust network parameters. This reduced the need for power-intensive methods such as GPS by conserving energy and enhancing the precision of node positioning within the network.

Key findings from the literature can be summarized in Table 8.

**Figure 7:**
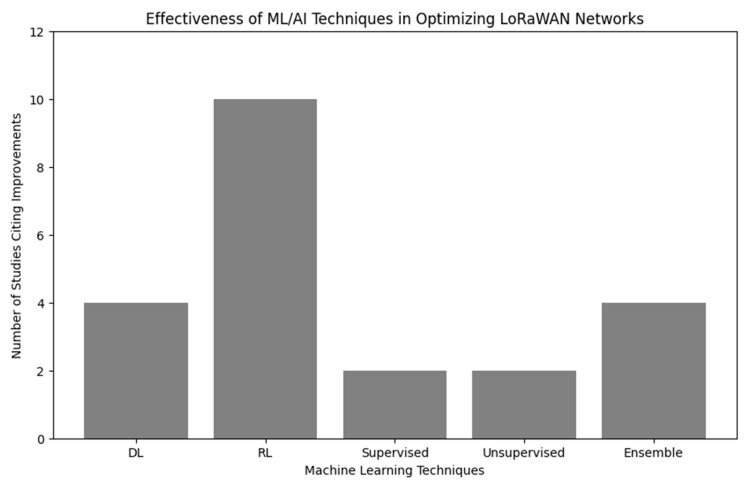
Effectiveness of ML/AI Techniques.

A visual representation of the most effective techniques discussed in literature can be seen in Figure 7.

RL emerged as the most frequently adopted ML technique for optimizing resource constraints and enhancing EE in LoRaWAN networks, followed by DL and Ensemble Learning. Supervised and Unsupervised Learning techniques, while effective, are mentioned less frequently in this specific context.

### 5.3. RQ3: What Are the Parameters That Are Optimized to Enhance the Performance of LoRaWAN Networks?

Research in the optimization of LoRaWAN networks focused mainly on enhancing network performance by adjusting various parameters like SF, TP, and Duty Cycle (DC) among others. All the studies in review have used various methodologies to achieve significant improvements in network efficiency, energy consumption, and overall reliability. The key findings from the literature can be summarized as follows in Table 9.

**Figure 8:**
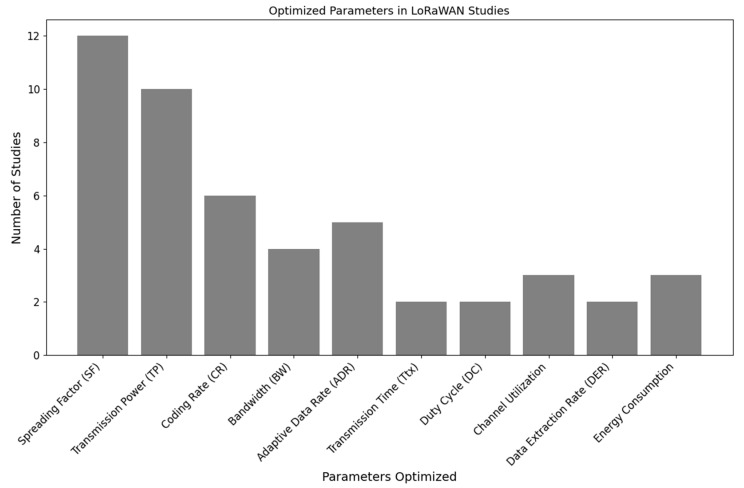
Optimized Parameters in studies.

Findings suggested that TP, SF, CR, ADR, and BW were addressed and optimized by most of the studies, as shown in Figure 8. As TP and SF are crucial for enhancing network efficiency and coverage, the DL techniques, along with RL, have achieved optimal results and ensured minimum energy usage, as discussed in [32,36,37]. ADR has been optimized to reduce packet loss along with CR to enhance data transmission, as in [33,35,38,40] where authors have used DL, RL, and ML to predict the adapting rates to improve error resilience and network reliability. Effective channel management is also essential for minimizing collisions and maximizing throughput. Authors in [41,47] have used multi-agent approaches that allow dynamic adaptation to network conditions. It has significantly improved PDR and overall network performance. The optimization of these parameters has significantly improved overall network performance.

### 5.4. RQ4: What Gaps and Opportunities Exist for Future Research in the Field of LoRaWAN Performance and Energy Efficiency Optimization?

LoRaWAN technology faces several persistent and emerging challenges that impact its efficiency and scalability. Various studies have highlighted critical gaps that offer opportunities for substantial research and development.

#### 5.4.1. Interference and Congestion Management

In the studies [30,41], the authors have focused on the challenge of managing interference and network congestion, particularly in dense deployments. The author [51] stated the impact of interference and collision when multiple LoRa devices are involved, especially in dense network deployments. LoRaWAN operates on unlicensed frequencies, and that leads to a high risk of collision and interference from both LoRa and non-LoRa sources. The studies emphasized the need for more effective solutions to mitigate interference, which could include developing advanced ML algorithms that dynamically adjust network parameters to optimize traffic flow and reduce collision rates. For instance, the author [30] suggests exploring multi-agent systems for coordinated resource allocation, which could significantly improve the network’s resilience to interference.

#### 5.4.2. Energy Efficiency and Optimization

Energy consumption remains a critical issue in LoRaWAN networks, especially because of the typical deployment of battery-operated devices over extensive areas. Studies [38,50] highlighted the gap in creating more efficient energy management strategies. The study by [52] discussed gaps in LoRaWAN performance and EE optimization. The author suggested addressing the issues caused by LoRaWAN’s Aloha-like nature, which affects the network’s reliability under high-traffic and large-scale deployments. These could involve refining existing models to more accurately predict energy consumption based on a variety of operational conditions or developing new technologies that minimize energy usage without compromising the network’s efficiency. The author’s [50] work, using Markov Decision Processes for dynamic adaptation, points to the potential of using sophisticated modeling to enhance energy predictions and device lifespan estimations.

#### 5.4.3. Adaptability to Environmental and Operational Variabilities

The adaptability of LoRaWAN to diverse environmental conditions is another gap identified in [30,42,44]. These studies have highlighted the importance of developing adaptive algorithms that automatically adjust to changing environmental conditions, such as weather variations and urban topographical changes, which affect signal propagation and network performance. There is also a need to enhance indoor localization accuracy [35]. Current methods have limitations in differentiating signals accurately across closely spaced indoor environments. Traditional path loss and shadow fading models do not account for various environmental effects, which can significantly impact LoRaWAN’s performance [52]. The author [41] stated a gap that existed in adapting to dynamically changing underground environments. The main challenges included the difficulty in managing massive data collisions and the constraints imposed by the underground setting on EE. Mobile IoT applications, such as asset tracking, introduce challenges related to rapid changes in signal strength because of their mobility and environmental conditions [34]. The author [28] specifically suggested integrating more diverse environmental data into predictive models to enhance network reliability and efficiency under different climatic conditions.

**Figure 9:**
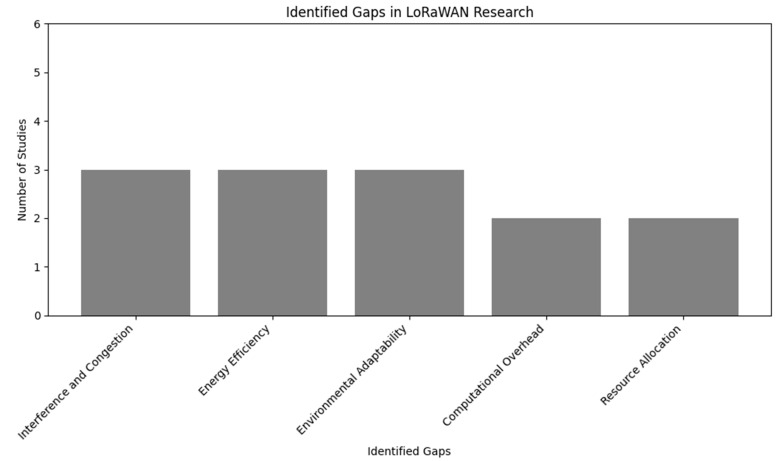
Gaps identified by literature.

Figure 9 shows the frequently identified gap across the literature. Despite the advancements, managing interference and congestion, particularly in dense network deployments, remains a significant challenge. There is a need for more sophisticated machine learning models that can dynamically adjust network parameters to mitigate interference impacts effectively.

#### 5.4.4. Opportunities and Future Research

Authors in several studies have devised various future work directions to enhance the performance of LoRaWAN networks.

The author [34] suggested that existing ADR mechanisms like BADR (Blind ADR) and standard ADR do not effectively address massive packet loss in dynamic and mobile environments due to inadequate SF adjustments. They suggested a need for more sophisticated algorithms that can dynamically and accurately adjust SF based on real-time changes in the network environment. Future research could focus on developing models that better understand and predict the effects of mobility on signal degradation and network performance. Future work could focus on extensive field tests to validate the effectiveness of AI-based resource allocation strategies under diverse and unpredictable environmental conditions.

To overcome computational overhead and complexity, there’s a need for simplified algorithms that minimize computational overhead by using predictive analysis to predict potential re-transmission and maintain network performance, especially in resource-constrained environments [50]. There is a need for improved methods of online resource allocation based on application constraints, which has not yet been fully explored despite its significance in real-world deployments. The authors [52] suggested that integrating a multi-agent system for resource allocation could significantly enhance the scalability and efficiency of LoRaWAN networks by customizing and designing semi-automated ML models. Prediction analytics is also highlighted in the study [31], and the author stated that we need more robust ML models that can handle high error rates and predict network behavior more accurately. An opportunity to develop better clustering algorithms that can more effectively categorize devices based on their behavior and operating conditions is also discussed [31]. To mitigate the impact of inference and collision, the authors [51] suggested evaluating LoRaWAN’s performance over distances greater than 100 m, particularly in industrial environments. There is also a need for sophisticated simulation models that integrate real-world geographical data to enhance the precision of network planning. As gaps remain in achieving optimal gateway deployment and managing transmission parameters effectively across varying urban topographies [42]. Future research could focus on refining ML algorithms and the use of real-time data [51] for better prediction and management of network behaviors and developing more dynamic and adaptive network configurations to accommodate changing urban landscapes and IoT demands. Further exploration of energy-saving technologies and methodologies to extend the life of IoT devices in the network could also provide substantial benefits in improving the efficiency of LoRaWAN deployments [30,41]. Table 10 summarizes the key findings from the study to answer RQ4.

### 5.5. Discussion

It has been observed while exploring the literature on optimization of LoRaWAN networks that recent research has highlighted the adoption of advanced ML and AI techniques to address key challenges such as EE, resource allocation, and network robustness. The reviewed studies have utilized a range of methods, from DL and RL to ensemble learning, which shows that there are broad and evolving methodologies that aim to enhance the performance of LoRaWAN systems across various operational environments. One of the common areas of focus has been improving EE. Energy efficiency is a critical consideration for networks mainly powered by battery-operated devices. Techniques such as dynamic priority scheduling and ML algorithms like Gradient Boosting and RF have been used to intelligently manage network resources, which has reduced power consumption while maintaining and enhancing network performance. For instance, it has been seen that the utilization of DRL has demonstrated effective results in optimizing transmission power and spreading factors in real-time, which are important for adapting to network density and transmission requirements dynamically.

Moreover, the capability of these networks to adapt to environmental variables has also been observed as a significant advancement. Those ML models, which specifically used supervised learning, have been proficient at predicting and adapting to changes in network conditions, which is essential for maintaining connectivity and efficiency in diverse settings, from urban centers to underground facilities. This integration of environmental adaptability highlights the potential of AI to bring LoRaWAN into more complex and demanding applications, such as IIoT and smart cities, where diverse and complex network environments exist. The literature also discussed the use of datasets and various network simulators for the evaluation of these models. It has been observed that strategic use of both real-world data and simulated environments has emerged as a crucial approach for advancing LoRaWAN technology. Various authors have used real-world as well as simulated datasets to evaluate the ML and DL models. The adoption of standard simulation platforms such as NS-3, LES, and others allowed researchers to model complex network scenarios and test hypotheses in controlled settings. These simulations are crucial for understanding potential network behaviors under diverse conditions that might not be available or practical to test in a real-world setup due to constraints on resources, time, or environmental control.

Despite these advancements, the literature has also revealed ongoing challenges, such as managing interference and congestion in densely deployed networks. The studies stated a need for more advanced multi-agent systems and enhanced learning algorithms that can operate and adapt to the restrictions of the unlicensed spectrum used by LoRaWAN. These systems must not only manage internal network conditions but also dynamically mitigate external interference, which remains a notable challenge for reliable connectivity. While the body of work on LoRaWAN optimization through AI and machine learning is evolving and showing substantial benefits, it also highlighted clear directions for future research. This included the development of algorithms that can further minimize energy consumption, enhance adaptive responses to environmental and network changes, and improve interference management. These areas are important for the scalability and sustainability of LoRaWAN implementations. Future studies should also focus on the integration of real-world testing to validate these models and obtain better results.

## 6. Conclusions

A systematic literature review has been conducted to evaluate the performance of LoRaWAN technology and how the use of ML and AI can enhance overall network efficiency. The SLR has highlighted the key role of advanced algorithms in optimizing resource allocation, managing network parameters dynamically, and adapting to diverse environmental conditions. It also addressed some of the critical challenges faced by LoRaWAN deployments. The review followed the PRISMA model for selecting studies that particularly focused on LoRaWAN and ML.

The extensive literature review has been conducted, and key findings have been observed and stated as follows:

- DRL, and supervised learning techniques have shown efficiency and improvements in refining energy consumption strategies.
- These methods have ensured robust network connectivity across diverse environments and enhanced the sustainability of LoRaWAN systems. They have also extended the operational life of battery powered IoT devices, which is a crucial factor for large-scale deployments.

Various key parameters are optimized to ensure better performance, specifically the SF. SF is crucial for determining communication range, data rate, and energy consumption. It determines how data is spread across the spectrum and has a direct influence on the signal’s robustness and network capacity. SF also influences network capacity and collision rates. In dense network deployments, the choice of SF can lead to increased collisions if not managed properly.

- Adaptive algorithms, including RL, are used in the literature to optimize the SF according to real-time network conditions to mitigate collisions and optimize network performance.
- Various sophisticated machine learning models have also been developed by the studies to dynamically adjust SF to reduce collision rates and improve overall network reliability.
- The adjustments in BW have also been addressed. It has impacted the data rate and energy consumption, where wider bandwidths increase the data rate but also raise the power requirements.
- In contrast, optimization of CR determines the redundancy in data transmission and enhances error correction capabilities at the cost of increased message length.

SLR has reviewed how the state-of-the-art has focused on dynamically balancing these parameters to optimize network throughput and reliability, especially in variable environmental conditions. Advanced algorithms, often in integration with SF adjustments, have dynamically optimized BW and CR to maintain optimal performance. This is crucial for adapting to network load and channel quality to minimize packet loss and improve link stability.

Future work will focus on reviewing real-time adaptive systems that use ML to dynamically adjust network parameters. Future work also aims to explore cross-layer design strategies that consider interactions between different layers of the network protocol stack to optimize overall network performance.

## Figures and Tables

**Table 1:** Performance overview and technical comparison [5,8,13].

Features	Sigfox	LoRaWAN	NB-IoT
Deployment Standardization	Proprietary	LoRa Alliance Standard since 2015	3GPP Standard
Frequency Bands	Unlicensed sub-GHz ISM bands	Unlicensed sub-GHz ISM band	Licensed frequency bands (GSM or LTE)
Modulation	BPSK in Ultra Narrow Band (100 Hz)	Chirped Spread Spectrum (LoRa)	QPSK in OFDMA (Downlink) and FDMA (Uplink)
Data Rate	Up to 100 bps	300 bps to 50 kbps	Up to 200 kbps (Downlink), 20 kbps (Uplink)
Payload Length	Up to 12 bytes	Up to 243 bytes	Up to 1600 bytes
Communication Classes	Uplink initially, later bidirectional	A (bidirectional), B, C	Standalone, Guard Band, In-Band
Maximum Range	Exceeding 40 km	Urban = 5 km, Rural = 20 km	<10 km (Urban), up to 10 km (Rural)
Maximum Devices per Cell	50k [8]	50k [8]	Over 100,000 [8]
Energy Consumption	Very low	Low to moderate	Low to moderate
Latency	Variable	Low to high, based on classes	Low due to synchronous communication
Scalability	Constrained	Highly scalable	Very scalable (Over 100,000 devices)

**Table 2:** Summary of the Literature Review.

Author (s)	Year	Technique Used	Problem Addressed	Gap Identified
Park et al. [36]	2020	Deep Reinforcement Learning (DRL)	Optimal transmission parameter distribution in LoRaWAN	Existing ADR MAX not achieving best throughput
Ilahi et al. [49]	2020	Deep Reinforcement Learning, Q-learning	Resource allocation in dense LoRa networks	Existing allocation strategies not supporting device mobility
Cuomo et al. [31]	2020	Machine Learning, LSTM, Decision Trees	Scalability and network performance in LoRaWAN	High error rates in devices not addressed effectively
Rajab, et al. [43]	2021	Support Vector Regression (SVR), DNN	Power consumption in IoT devices	Traditional methods not extending battery life sufficiently
Fedullo et al. [37]	2021	Reinforcement Learning (RL)	Reliability in high node density LoRaWAN	Traditional ADR not optimizing SF and transmission power effectively
Yazid et al. [38]	2021	Deep Q-Network (DQN), Reinforcement Learning	Node durability in LoRaWAN	Current methods do not effectively extend node lifespan
Farhad et al. [33]	2022	Deep Learning, Gated Recurrent Unit (GRU)	Resource management in LoRaWAN	Inadequate SF allocation causing packet loss
Farhad et al. [34]	2023	Deep Neural Networks (DNN)	Packet loss due to inefficient SF utilization	Insufficiency of existing ADR and BADR techniques
Perković et al. [35]	2023	Neural Networks (NN)	Indoor localization accuracy in LoRaWAN	Environmental factors causing variability in signals
Ossongo et al. [39]	2024	Federated Reinforcement Learning (FRL)	Network reliability for LoRaWAN connected objects	Existing models do not optimize device throughput efficiently

**Table 3:** Summary of DL and NN techniques.

Author (s)	Year	Technique Used	Model	Application
Tu et al. [32]	2020	DL	ANNs	Optimization of transmit power
Farhad et al. [33]	2022	DL-based Resource Allocation	GRU	Resource management in large-scale LoRa-enabled device deployments
Farhad and Pyun [34]	2023	DNN AI-ERA approach	DNN	resource assignment issue in static and mobile IoT applications
Perković et al. [35]	2023	ML	NN	Indoor localization within LoRaWAN

**Table 4:** Summary of RL techniques.

Author (s)	Year	Technique Used	Model	Application
Park et al. [36]	2020	DRL	Multiple DRL Agents	LPWA networks such as LoRaWAN, wireless communication environments for the IoT.
Fedullo et al. [37]	2021	RL	Stochastic Discrete Approach	Development of an adaptable LoRaWAN strategy for industrial applications.
Yazid et al. [38]	2021	DQN	Deep Q-Network (DQN)	Lifespan of the LoRa Class A end-nodes
Ossongo et al. [39]	2024	FRL and Network Slicing (NS)	Neural Model with 2 Hidden Layers	Adaptive resource allocation and prioritization in infrastructure networks.
Sandoval et al. [40]	2019	RL, Deep RL, Q-Learning, and SARSA	RL techniques	LoRa-based networks
Zhao et al. [41]	2023	Multi-agent RL	MARL	EE in WUSNs integrated with LoRaWAN

**Table 5:** Summary of ML and Supervised and Unsupervised learning techniques.

Author (s)	Year	Technique Used	Model	Application
Cuomo et al. [31]	2020	ML	k-means clustering, LSTM NN, DTs	Real large-scale LoRaWAN network
Garrido et al. [52]	2023	Supervised Learning	ML multi agent approach	EE and network performance in LoRaWAN with Clock skew estimation
Piechowiak et al. [42]	2023	ML	ML clustering (K-means)	A LoRaWAN network infrastructure in smart cities.
Rajab et al. [43]	2021	ML	SVR, DNN	Long distance Network, LPWANs connected to the IoT
Guerra et al. [44]	2024	ML and DL	ARIMA, ANN, SVM	Accuracy in LoRaWAN in outdoor devices
Yatagan et al. [25]	2019	Supervised Learning	Decision Tree Classifier (DTC), SVM	Large geographical area in LPWANs
Asad Ullah et al. [45]	2019	K-means clustering-based approach	K-means clustering	Large-scale LoRa networks

**Table 6:** Summary of ML ensemble techniques.

Author (s)	Year	Technique Used	Model	Application
Minhaj et al. [30]	2023	Supervised ML, RL	EXP4, Lasso algorithm	Large scale IoTs
Aihara et al. [47]	2019	ML based Q-learning	Q-learning	Large coverage areas in LoRaWAN
Pandangan et al. [48]	2020	Ensemble Learning	KNN-RFR	Outdoor localization accuracy in LPWAN technologies
Ilahi et al. [49]	2020	DRL, cognitive radio systems	DRL	Mobility in dense LoRa networks (Mobile End Devices)
Teymuri et al. [50]	2023	RL, Multi-Armed Bandit	LP-MAB	IoT and LPWANs, specifically LoRaWAN
Kaur et al. [51]	2022	ML	ANN, PSO	Industrial IoT applications.
Palacio et al. [52]	2023	ML	MLR, ANNs	Practical IoT applications
Alenezi et al. [46]	2020	Dynamic Transmission Priority Scheduling	Naive Bayes, unsupervised clustering	Dense applications in LoRaWAN

**Table 7:** Summary of datasets and simulators.

Author (s)	Year	Dataset Features and Details	Dataset Size	Simulation Details
Farhad et al. [34]	2023	Generated using ns-3 simulator. X, Y coordinates, P_rx_, SNR, ACK status. 500 EDs, 10 days, packets every 10 min	500 end devices, multiple data points per device over 10 days	NS-3 simulator with long-distance propagation, shadowing, and interference models
Alenezie et al. [46]	2020	Dense network up to 1000 nodes. Nodes transmit with SF7. Data included sensor readings.	Up to 1000 nodes within a 3 km² area	NS-3, focused on dynamic transmission PST using K-Means clustering
Farhad et al. [33]	2022	Generated using ns-3 simulator. X, Y coordinates, P_rx_, SNR, ACK status. Each ED transmits six uplink packets hourly	500 EDs, frequent data collection	NS-3 with a GRU model to dynamically allocate SFs
Rajab et al. [43]	2020	Derived from TTN UK formulas. Main power consumption factors from 20 reduced to 15	35,192 entries	Data Loader Techniques. Data scaling (min-max normalization)
Cuomo et al. [31]	2020	Real LoRaWAN network in Italy. Data from water metering service including SF, SNR, RSSI, etc.	372,119,877 packets, 290 water meters 89,528 EDs	Real-world data analysis
Ossongo, et al. [39]	2024	Generated from network interactions, realistic IoT conditions	SF, TP, BW, CR, Max 1000 nodes per slice	LoRaSim, open-source environment in Python
Park et al. [36]	2020	Data generated from network interactions	30 LoRa nodes	Custom simulation includes one gateway. 5 groups and 1500 × 1500 m^2^ topology size
Sandoval et al. [40]	2019	Packet rate (0.01 to 2 packets/s), Packet importance (0–1), SNR (−23 to 23 dB), Transmission power (14 dBm), Packet length (15–30 bytes)	Networks of 20 to 200 nodes	Simulated using SimPy on an 8-core Intel Xeon server, 100 different seeds, twelve-hour simulations
Guerra et al. [44]	2024	LoRaWAN RSSI measurements	2029 records reduced to 1870 records	Forecasting using ARIMA
Pandangan et al. [48]	2020	Open access LoRaWAN dataset	Number of Messages 130,430 collected in Antwerp, Belgium	Computational model

**Table 8:** Efficiency of AI and ML approaches.

Author (s)	AI/ML Technique	Improvement
Tu et al. [32]	DL ANN	Enhanced EE through optimal TP management
Alenezie et al. [46]	K-Means Clustering	Improved network scalability while maintaining low packet collision rates and enhanced EE.
Farhad et al. [33]	GRU DL	11% increase in packet success ratio
Farhad and Pyun [34]	DNN	Improved SF management and packet success rates
Perkovic et al. [35]	NN	Reduced energy consumption, 98% localization accuracy
Park et al. [36]	DRL	15% improvement in throughput
Fedullo et al. [37]	RL	10% improvement in DER
Yazid et al. [38]	DQN	Extended node lifespan by optimizing transmission parameters
Zhao et al. [41]	MARL	Optimized EE better than traditional ADR
Cuomo et al. [31]	LSTM, DT, k-means	Predicted SF impacts with 3.5% error rate
Kaur et al. [51]	ANN, PSO	Enhanced BER, spectral efficiency, and outage probability
Ossongo et al. [39]	Federated RL, Network Slicing	Optimized throughput by network slicing, achieved 3% rejection rate
Sandoval et al. [40]	Q-Learning, SARSA	147% increase in throughput
Gonzalez-Palacio et al. [52]	ML-based CPLS models	43% maximum energy improvement in indoor localization
Garrido et al. [20]	ML multi-agent methodology	30% reduction in power consumption and improved network size
Rajab et al. [43]	SVR, DNN	Extended battery life and reduced power consumption
Minhaj et al. [30]	Supervised ML, RL	Ten times faster convergence in SF and TP allocation
Aihara et al. [47]	Q-Learning	20% improvement in PDR compared to random allocation
Pandangan et al. [48]	Ensemble Learning (kNN, RFR)	16% and 29% improvements in mean and median error respectively
Ilahi et al. [49]	DRL	500% improvement in PDR, resistant to frequency jamming
Teymuri et al. [50]	RL, MAB	Consistently outperformed previous approaches in EC and PDR
Piechowiak et al. [42]	ML Clustering	Significant decrease in energy consumption with improved coverage
Asadullah et al. [45]	K-Means Clustering	Improved network coverage probability by up to 5% and enhanced worst-case node performance by 1.53 times
Guerra et al. [44]	ARIMA, ANN, SVM, RF, LSTM, Hybrid (ARIMA-ANN, etc.)	Improved forecasting of RSSI ARIMA with temperature as regressor showed competitive performance and significant accuracy.
Yatagan et al. [25]	SVM and Decision Tree Classifier	Improved PDR and reduced collisions by optimizing SF assignment

**Table 9:** Optimization parameters to enhance performance.

Author (s)	Parameters Optimized	Optimization Focus	Method of Optimization
Alenezi et al. [46]	Transmission Priority and Intervals, SF, TP and Time-on-Air, Packet Size, Energy Consumption, Collision Rate	Reducing packet collision rates, transmission delays, and energy consumption by managing transmission priorities, SFs, and power levels.	Dynamic PST, Unsupervised Learning
Farhad et al. [34]	SF, TP, Packet Success Ratio, Energy Consumption	Optimizing dynamic resource allocation, improving packet success ratios, and minimizing energy usage.	AI-ERA
Teymuri et al. [50]	SF, TP, Carrier Frequency (CF), Coding Rate	Enhancing network coverage, reducing noise resistance, and optimizing power use to improve overall network performance.	LP-MAB
Garrido et al. [52]	SF, T_tx_, DC Network Synchronization and Scheduling Entities (NSSEs)	Adjusting transmission times, duty cycles to improve network synchronization and reduce energy consumption.	Multi-Agent Systems
Cuomo et al. [31]	SF, Inter-arrival Time (IT), ER, RSSI and SNR	Profiling and prediction to dynamically adjust network parameters for improved efficiency and performance.	ML Supervised and Unsupervised Learning
Kaur et al. [51]	Received Power, SF, TP, Outage Probability, BER, Spectral Efficiency	Optimizing received power and transmission parameters to maximize link performance and network efficiency.	PSO, ANN
Rajab et al. [43]	Tp, DC, modulation	Reducing power requirements through careful scheduling and power management.	Multi-Agent Systems
Ilahi et al. [49]	SF, Channel Frequency, Transmission Power, Airtime	DRL used to adaptively adjust PHY-layer parameters for improved network capacity and device mobility support.	DRL (Double Deep Q-Network)
Piechowiak et al. [42]	Number of Gateways, Transmission Parameters, SF, Energy Consumption	Strategic planning of gateway deployment and transmission settings based on real geographic data to optimize coverage and reduce energy usage.	ML
Tu et al. [32]	TP, SF, CR, BW	Reducing energy consumption and improving transmission reliability through adjusting several parameters.	DL
Gonzalez et al. [52]	TP, Path Loss and Shadow Fading, Environmental Variables, ADR	Emphasis on transmission power settings based on environmental conditions. Enhanced network reliability and EE.	ML ensemble Modeling
Aihara et al. [47]	Resource Allocation, PDR, CSMA/CA, Interference Management	Efficient assignment of frequency channels and improvement in PDR through dynamic adjustments of network parameters to minimize packet collisions and manage interference.	Q-learning
Ossongo et al. [39]	TP, SF	Dynamic adjustments of TP and SF to optimize efficiency in network slices. Focused on EE and collision probability.	FRL
Park et al. [36]	SF, TP, Channel Allocation	Optimization of communication parameters to balance energy consumption, data rate, and reduce interference.	DRL
Guerra et al. [44]	RSSI	Forecasting RSSI using weather parameters and employing hybrid models to improve prediction accuracy.	ARIMA, AI Techniques
Sandoval et al. [40]	SF, Time on Air, Transmission Duty Cycle (TDC)	Optimizing communication parameters to improve EE and resource usage.	ML, RL
Ilahi et al. [49]	SF, CR, BW	Resource allocation and EE in dense LoRa networks	DRL
Asadullah et al. [45]	SF	Optimizing the allocation of SF to improve network coverage, reliability, and fair resource distribution in large-scale LoRa networks.	K-means clustering
Minhaj et al. [30]	SF, TP, CR	Dynamic optimization of SF, TP, and CR to enhance transmission range, power efficiency, and data transmission robustness.	ML and RL
Fedullo et al. [37]	DER, SF, TP, Physical layer Parameters	Focusing on maximizing DER, balancing SF, and optimizing TP in industrial environments.	RL (SARSA)
Perkovic et al. [35]	SF, TP, DC, Channel Utilization	Tuning of SF, TP, duty cycle, and channel utilization for better network efficiency.	ML
Yazid et al. [38]	ADR, SF, TP, Channel Utilization	Dynamic adjustment of ADR, SF, and channel utilization to enhance network scalability.	ML
Pandangan et al. [48]	SF, TP, Channel Utilization	Optimizing SP and channel utilization to manage transmission efficiency and reduce collision rates.	ML
Zhao et al. [41]	TP, SF, ADR	Enhancing TP, SF and ADR for improved EE in underground sensor networks.	Multi-Agent RL
Yatagan et al. [25]	SF, TP, Channel Allocation	Optimizing SF, TP, and channel allocation to reduce time on air and improve EE.	DRL

**Table 10:** Gaps and opportunities in literature.

Author (s)	Gap Identified	Details	Future Directions
[16,27,37]	Interference and Congestion Management	High risk of collision in dense deployments	Develop advanced ML algorithms, explore multi-agent systems
[24,36,39]	EE and Optimization	Inefficiency under high-traffic and large-scale deployments	Refine energy models, develop new technologies for minimal energy use
[16,28,30]	Adaptability to Environmental and Operational Variabilities	Inadequate response to environmental changes affecting signal propagation	Develop adaptive algorithms, integrate diverse environmental data
[20,36]	Computational Overhead and Complexity	High computational overhead in dynamic environments	Develop simplified algorithms, use predictive analysis for re-transmission prediction
[17,39]	Online Resource Allocation and ML Utilization	Need for more effective device categorization and behavior prediction	Enhance clustering algorithms, integrate real-time data for ML models
